# A novel occludin-targeting monoclonal antibody prevents hepatitis C virus infection *in vitro*

**DOI:** 10.18632/oncotarget.24742

**Published:** 2018-03-30

**Authors:** Ken Okai, Naoki Ichikawa-Tomikawa, Akira C. Saito, Tetsuya Watabe, Kotaro Sugimoto, Daiki Fujita, Chikako Ono, Takasuke Fukuhara, Yoshiharu Matsuura, Hiromasa Ohira, Hideki Chiba

**Affiliations:** ^1^ Department of Basic Pathology, Fukushima Medical University School of Medicine, Fukushima, Japan; ^2^ Department of Gastroenterology, Fukushima Medical University School of Medicine, Fukushima, Japan; ^3^ Department of Molecular Virology, Research Institute for Microbial Diseases, Osaka University, Osaka, Japan

**Keywords:** tight junction, OCLN, HCV, entry factor, hepatocyte, Pathology

## Abstract

Since hepatitis C virus (HCV) is thought to enter into host hepatocytes using the same cellular pathways regardless of the genotypes, the host factors are promising targets to prevent and treat HCV infection. Human occludin (hOCLN) is one representative entry factor, and its second extracellular loop (EC2) contributes to the species selectivity of HCV-susceptibility. However, the exact function of hOCLN during HCV entry remains unknown, and no hOCLN-targeting antibodies or synthetic drugs that prevent and treat HCV infection have yet been developed. Here we generated the anti-hOCLN-EC2 monoclonal antibody (mAb) 67-2, and demonstrated that it efficiently inhibited HCV infection in the HCV-permissive human cell line Huh7.5.1. We also showed, using three different culture systems of Huh7.5.1 cells, that this novel mAb is accessible to OCLN from the basolateral side of hepatocytes but not from the apical side. In addition, our Western blot analyses indicated that the established 67-2 mAb reacted not only with hOCLN but also with mouse OCLN, strongly suggesting that 67-2 does not recognize the human-specific amino acids in OCLN-EC2. Moreover, we revealed that the anti-hOCLN-EC2 mAb 67-2 showed no adverse effects on cell viability or the barrier function of tight junctions.

## INTRODUCTION

Chronic infection with hepatitis C virus (HCV) affects an estimated 180 million individuals globally (approximately 3% of world's population), and is the leading cause of chronic hepatitis, liver cirrhosis and hepatocellular carcinoma [1, 2]. Recent advances in the development of direct-acting antivirals (DAAs), which target the viral life cycle in host cells, have extensively improved the efficacy of HCV treatment [3–5]. Despite the outstanding cure rates, a substantial number of patients do not respond to DAAs depending on the regimen and the HCV genotypes/subtypes/quasispecies, and the appearance of numerous DAA-resistant HCV variants has been reported [6–10]. In addition, DAAs are not able to prevent HCV infection, and no protective HCV vaccine is yet available.

Hepatocytes exhibit a specialized “hepatic polarity”, and their plasma membranes are divided into apical/canalicular and basolateral/sinusoidal domains by tight junctions. HCV initially interacts with the basolateral surface of hepatocytes, and is considered to enter at tight junctions via clathrin-mediated endocytosis irrespective of the HCV genotypes [11–13]. Therefore, the host factors that are involved in HCV entry are promising targets to prevent and treat HCV infection. Among the HCV entry factors, the scavenger receptor type B class I (SCARB1) [14], the tetraspanin CD81 [15] and the tight-junction proteins claudin-1 (CLDN1) [16] and occludin (OCLN) [17, 18] are at least required for HCV-susceptibility to host cells. Of these, CD81 and OCLN are responsible for the species specificity of HCV-susceptibility, and their human origin is necessary for promoting HCV permissivity to rodent cells both *in vitro* and *in vivo* [17, 19, 20]. Nevertheless, the mechanisms by which HCV interacts with these host factors and is eventually internalized into hepatocytes remain poorly understood.

OCLN, a tetraspan membrane protein with two extracellular loops, a short intracellular turn, and N- and C-terminal domains, is exclusively localized at tight junctions [21]. Among these domains, the second extracellular loop (EC2) accounts for species selectivity of OCLN to mediate HCV entry [17, 18]. In addition, six of 48 amino acids in the EC2 of OCLN differ between human and mouse, and at least two alanine residues (A223 and A224) are critical for the HCV-sensitivity to host cells [22]. Moreover, HCV triggers endocytosis of OCLN with viral proteins and their accumulation in endoplasmic reticulum [23]. However, the precise role of OCLN during HCV entry has not been defined.

We show in the present work the development of an anti-OCLN monoclonal antibody (mAb) that efficiently prevents HCV infection *in vitro*. We also provide evidence showing that the novel mAb is accessible to OCLN from the basolateral side of hepatocytes but not from the apical side. Furthermore, we demonstrate that this mAb exhibits no adverse effects on cell viability and the barrier function of tight junctions.

## RESULTS

### Generation of novel mAbs against hOCLN-EC2

**Figure 1:**
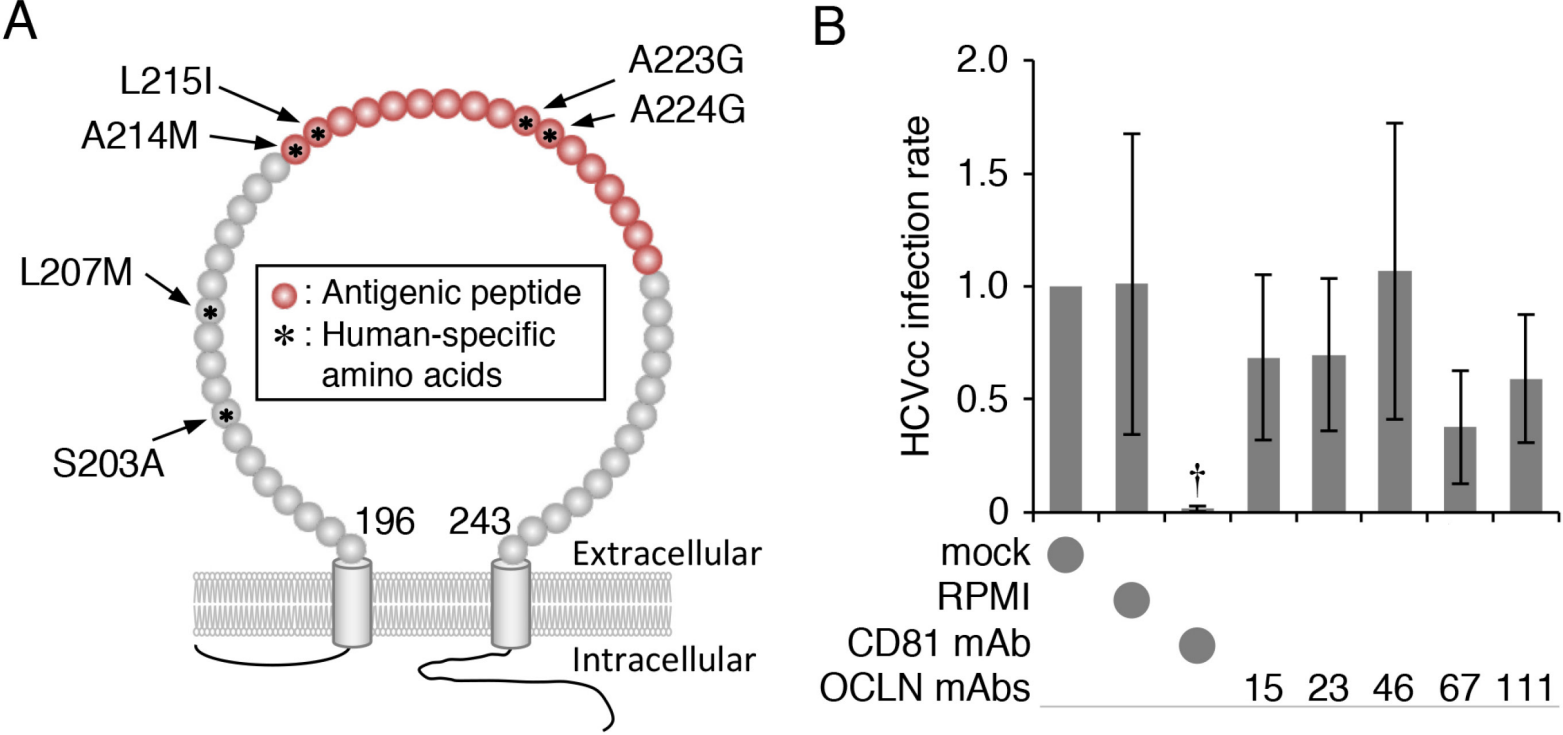
Generation of hOCLN-targeting mAbs (**A**) Schematic representation of the EC2 region of hOCLN protein. Antigenic peptide is shown in red. Asterisks indicate amino acids that are different between human and mouse. (**B**) Huh7.5.1 cells grown in monolayer were exposed to HCVcc and either mock, RPMI, anti-CD81 mAb (2.5 μg) or anti-OCLN-EC2 mAbs (100 μl of culture media), and total RNA from the cells was subjected to real-time RT-PCR analysis for the NS5A expression. The infection levels were expressed relative to the amount of the mock-treated cells, which was taken as 1. All values represent the mean ± SD (error bars; *n* = 5). Statistic values were determined compared with those in the RPMI-treated cells; ^†^*P* < 0.05.

We first generated mAbs against hOCLN-EC2 using the polypeptide corresponding to the amino acid sequence 214–230, which contains four human-specific amino acids, as an antigen (Figure 1A). The hybridomas were screened based on their ability to bind to the antigenic peptide, and five of the thirty-two positive clones were further analysed. Although these clones (clones 15, 23, 46, 67 and 111) exhibited a high affinity to the antigenic peptide (data not shown), they hardly prevented HCVcc (cell cultured HCV) infection of Huh7.5.1 monolayers compared with those treated with anti-CD81 mAb (Figure 1B). We, therefore, speculated that the anti-hOCLN-EC2 mAbs were not able to access to OCLN from the apical side of Huh7.5.1 monolayers.

### The anti-hOCLN mAb 67-2 prevents HCVcc infection in double-chamber culture system

**Figure 2:**
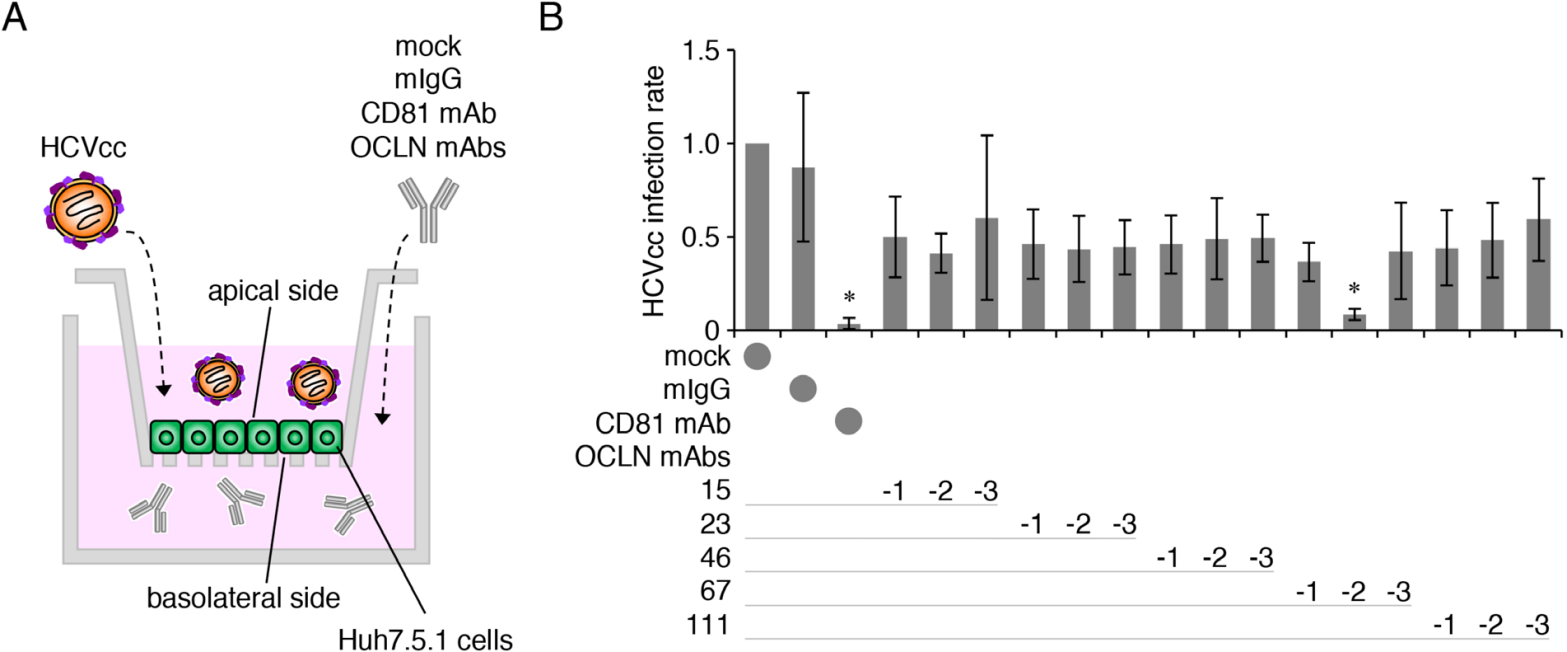
The anti-OCLN mAb 67-2 prevents HCV infection in double-chamber culture system (**A**) Illustration of Huh7.5.1 cell culture system using double-chamber methods to verify HCV infection. (**B**) Huh7.5.1 cells grown in double-chamber system were exposed to HCVcc and either mock, mIgG (50 μg), anti-CD81 mAb (2.5 μg) or anti-OCLN-EC2 mAbs (50 μg), and total RNA from the cells was subjected to real-time RT-PCR analysis for the NS5A expression. The infection levels were expressed relative to the amount of the mock-treated cells, which was taken as 1. All values represent the mean ± SD (error bars; *n* = 5). Statistic values were determined compared with those in the mIgG-treated cells; ^*^P < 0.01.

We then verified HCVcc infection using Huh7.5.1 cells grown in double-chamber culture system (Figure 2A). Since the cells failed to be infected by HCVcc upon its basolateral addition, most probably due to the trap in the transwell membrane, HCVcc was applied from the apical side. When fifteen subclones, derived from the five clones, were applied from the basolateral side in the double-chamber system, the mAb 67-2, like anti-CD81 mAb, significantly blocked HCVcc infection (Figure 2B).

### The anti-hOCLN mAb 67-2 blocks HCV infection in 3D culture

**Figure 3:**
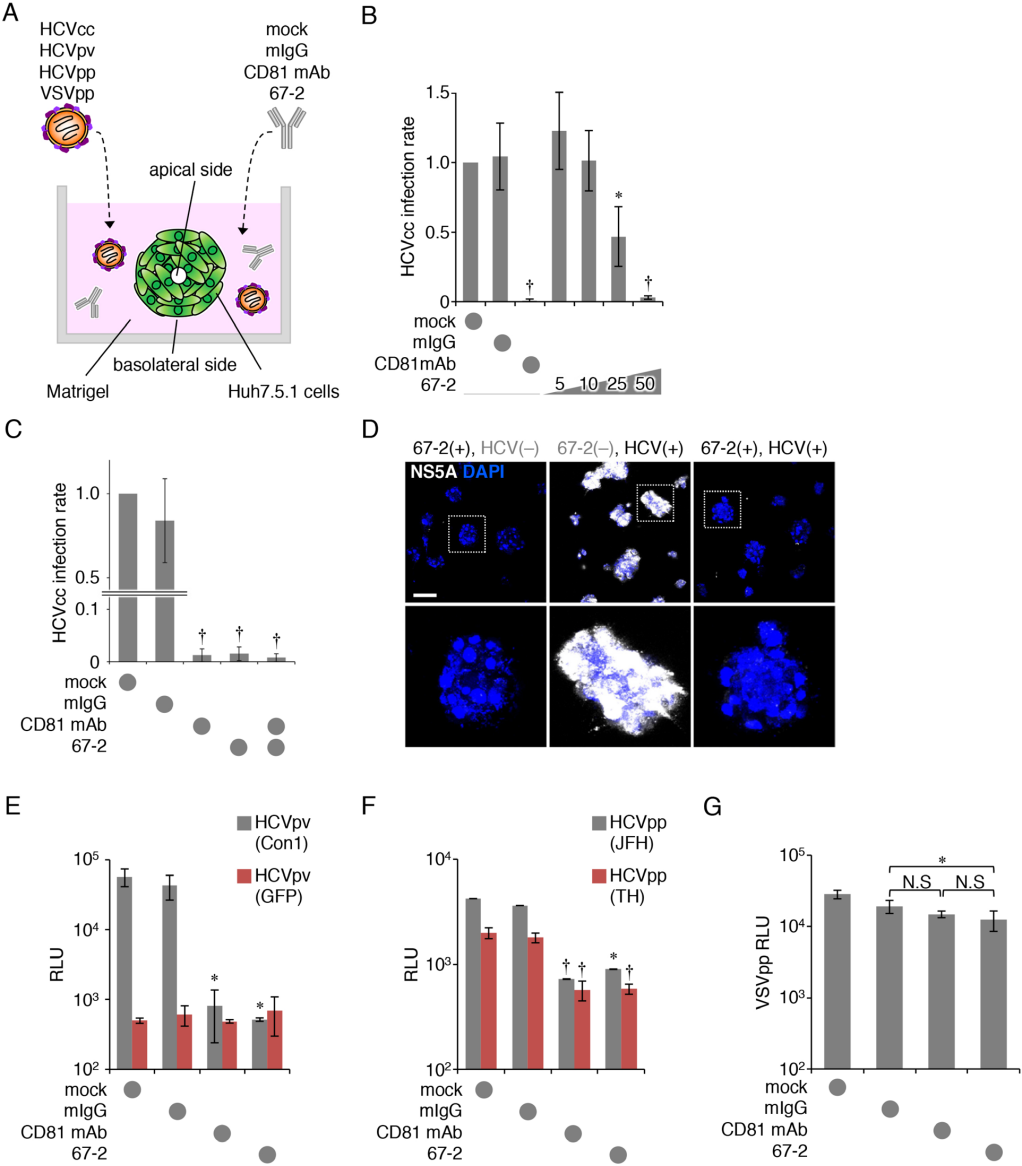
The anti-OCLN mAb 67-2 blocks HCV infection in 3D culture (**A**) Illustration of Huh7.5.1 cell culture system using matrigel-embedded 3D methods to verify HCV infection. (**B, C**) Huh7.5.1 cells grown in matrigel were exposed to HCVcc and either mock, mIgG (50 μg), anti-CD81 mAb (2.5 μg), the mAb 67-2 (50 μg) or both mAbs, and total RNA from the cells was subjected to real-time RT-PCR analysis for the NS5A expression. The infection levels were expressed relative to the amount of the mock-treated cells, which was taken as 1. (**D**) Huh7.5.1 cells grown in matrigel were treated with mIgG or 67-2 in the presence or absence of HCVcc, and were subjected to immunostaining for NS5A. Bar, 20 μm. (**E–G**) Huh7.5.1 cells exposed to mock, mIgG, anti-CD81 mAb or 67-2 in matrigel were subjected to infection assay for HCVpv (E), HCVpp (F) and VSVpp (G). Relative luminescence units (RLUs) were calculated based on the values of VSVpv-infected cells. All values represent the mean ± SD (error bars; *n* = 5 except for C [*n* = 6]). Statistic values were determined compared with those in the mIgG-treated cells; ^*^P < 0.01, ^†^*P* < 0.001. N.S, not significant.

We subsequently determined HCV infection using Huh7.5.1 cells grown in matrigel-embedded 3D cultures to mimic the environment of hepatocytes *in vivo* (Figure 3A). As shown in Figure 3B, the purified mAb 67-2, upon basolateral addition to matrigel-embedded 3D cultures, inhibited HCVcc infection in a dose-dependent manner (Figure 3B). The maximal effect of 67-2 on preventing HCVcc infection was similar to that of anti-CD81 mAb. In addition, these mAbs exhibited no synergistic effect on preventing HCVcc infection (Figure 3C). Immunostaining for NS5A also showed that 67-2 inhibited HCVcc infection in 3D culture (Figure 3D). Moreover, the basolaterally-applied 67-2 significantly prevented HCVpv (con1, genotype 1a) infection in 3D culture of Huh7.5.1 cells (Figure 3E). Besides, it efficiently blocked HCVpp (JFH1, genotype 2a; TH, genotype 1b) infection in 3D culture similarly to anti-CD81 mAb (Figure 3F). Although 67-2 minimally but significantly prevented VSVpp infection, the relative luminescence unit (RLU) was not significantly different from that in cells exposed to anti-CD81 mAb (Figure 3G), supporting that the inhibitory effect of the mAb 67-2 on HCVpv and HCVpp is not due to variations in infection rates.

### The mAb 67-2 barely inhibits HCVpv infection in Huh7.5.1 monolayer

**Figure 4:**
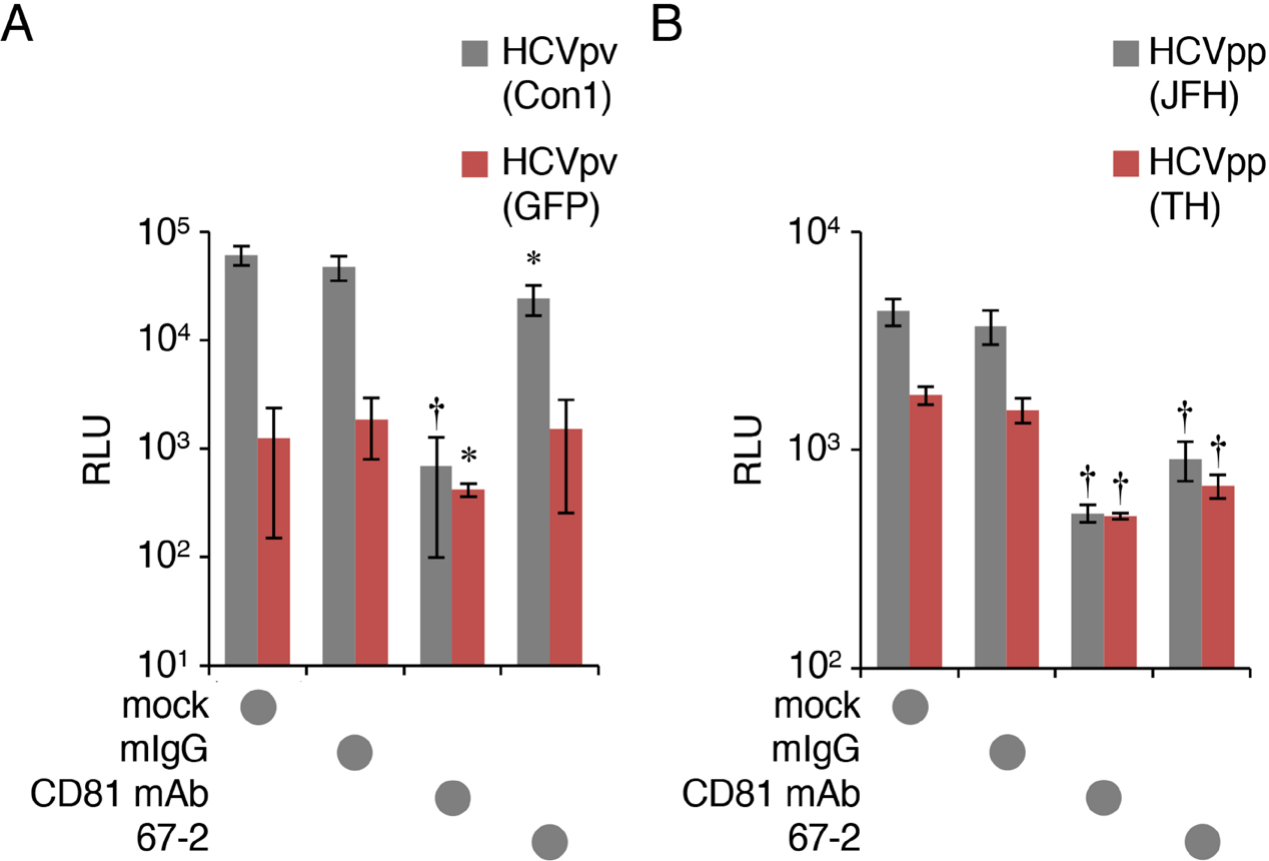
The mAb 67-2 hardly inhibits HCVpv infection in Huh7.5.1 (**A, B**) Huh7.5.1 monolayer exposed to mock, mIgG (50 μg), anti-CD81 mAb (2.5 μg) or 67-2 (50 μg), was subjected to infection assay for HCVpv (A) and HCVpp (B). Relative luminescence units (RLUs) were calculated based on the values of VSVpv-infected cells. All values represent the mean ± SD (error bars; *n* = 5). Statistic values were determined compared with those in the mIgG-treated cells; ^*^P < 0.05, ^†^*P* < 0.001.

We next evaluated HCVpv and HCVpp infection in Huh7.5.1 monolayers. As expected, the apically-applied 67-2 marginally prevented HCVpv infection in monolayer culture, and its effect was much less than that of anti-CD81 mAb (Figure 4A). Exposure of Huh7.5.1 monolayers to the 67-2 mAb resulted in significant reduction in infection with HCVpp; however, the effectiveness was weaker than that of anti-CD81 mAb (Figure 4B).

### The mAb 67-2 does not show cytotoxicity in Huh7.5.1 cells

**Figure 5:**
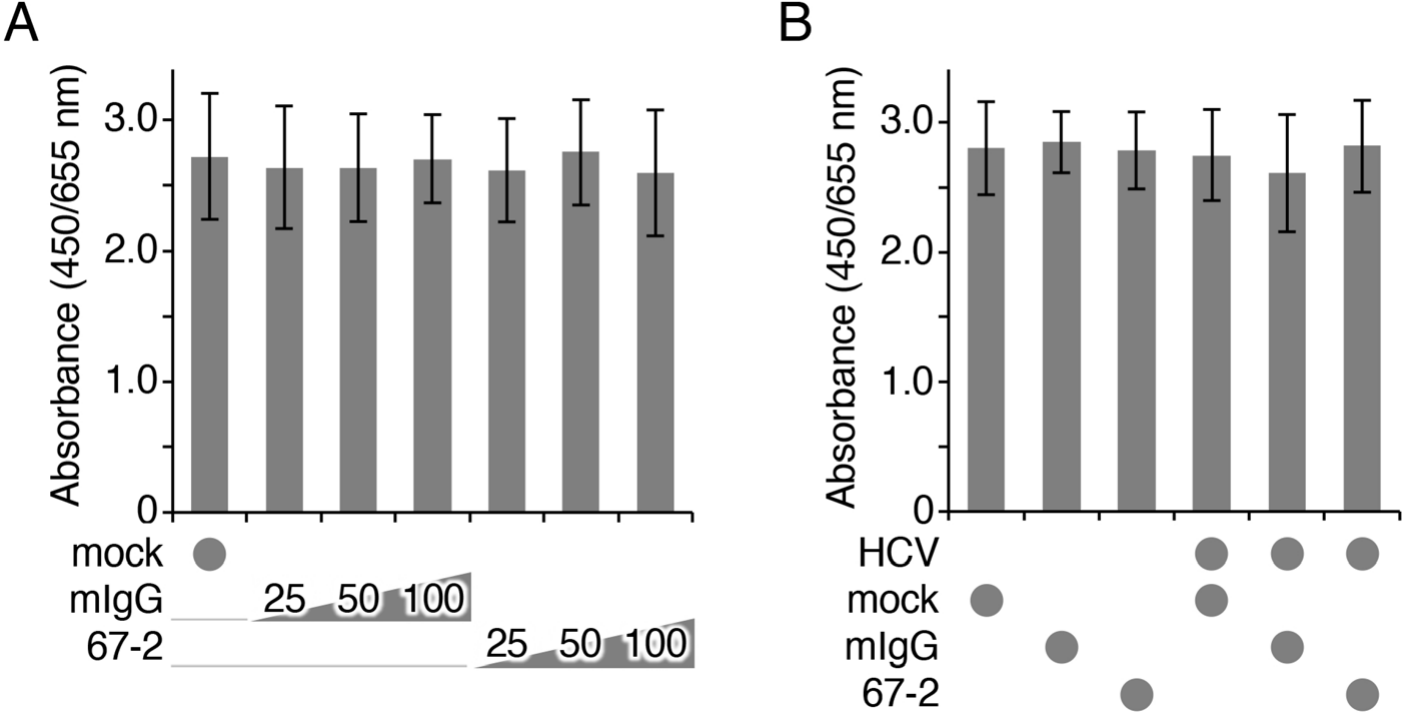
The mAb 67-2 does not affect cell viability of Huh7.5.1 3D culture (**A, B**) Huh7.5.1 cells grown in matrigel were exposed to the indicated treatments, and the cell viability was determined by XTT assay. All values represent the mean ± SD (error bars; *n* = 4).

To validate the safety of the 67-2 mAb *in vitro*, we subsequently clarified by XTT assay whether the 67-2 possessed cytotoxicity on Huh7.5.1 cells. As shown in Figure 5A, 67-2 did not affect cell viability of Huh7.5.1 in 3D culture at any concentration tested. In addition, 67-2 showed no cytotoxicity in the presence of HCVcc (Figure 5B).

### The mAb 67-2 cross-reacts with mouse OCLN

**Figure 6:**
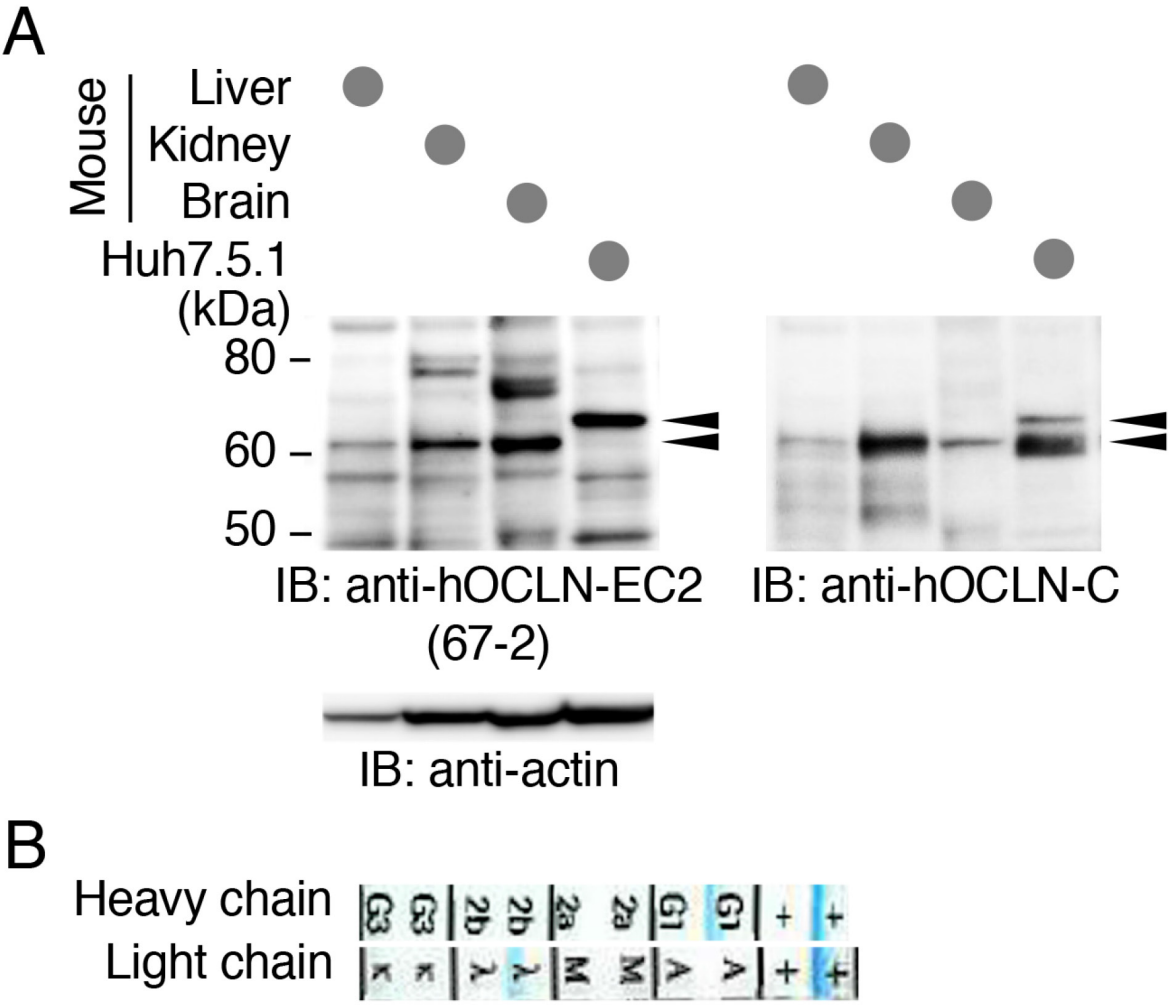
The mAb 67-2 recognizes human and mouse OCLNs (**A**) Total cell lysates from mouse liver, kidney and brain tissues as well as Huh7.5.1 cells were separated by SDS-PAGE and immunoblotted (IB) with 67-2, followed by chemiluminescence detection. The blot was stripped and reimmunoprobed with a mouse mAb against the C-terminal domain of hOCLN (anti-hOCLN-C) and an anti-actin Ab. Arrowheads show specific signals. The mobility of molecular mass markers (kilodaltons) is indicated on the left. (**B**) The subclass of the mAb 67-2 was determined by isotype analysis.

For future *in vivo* use of the anti-hOCLN-EC2 mAb 67-2, we then determined by Western blot whether it reacted with not only hOCLN, but also with mouse OCLN. As expected, 67-2 recognized human OCLN that was endogenously expressed in Huh7.5.1 cells (Figure 6A). It also reacted with mouse OCLN in the liver, kidney and brain tissues. Moreover, isotype analysis revealed that the subclass of 67-2 was IgG1λ (Figure 6B).

### The mAb 67-2 has no effect on the function of tight junctions

**Figure 7:**
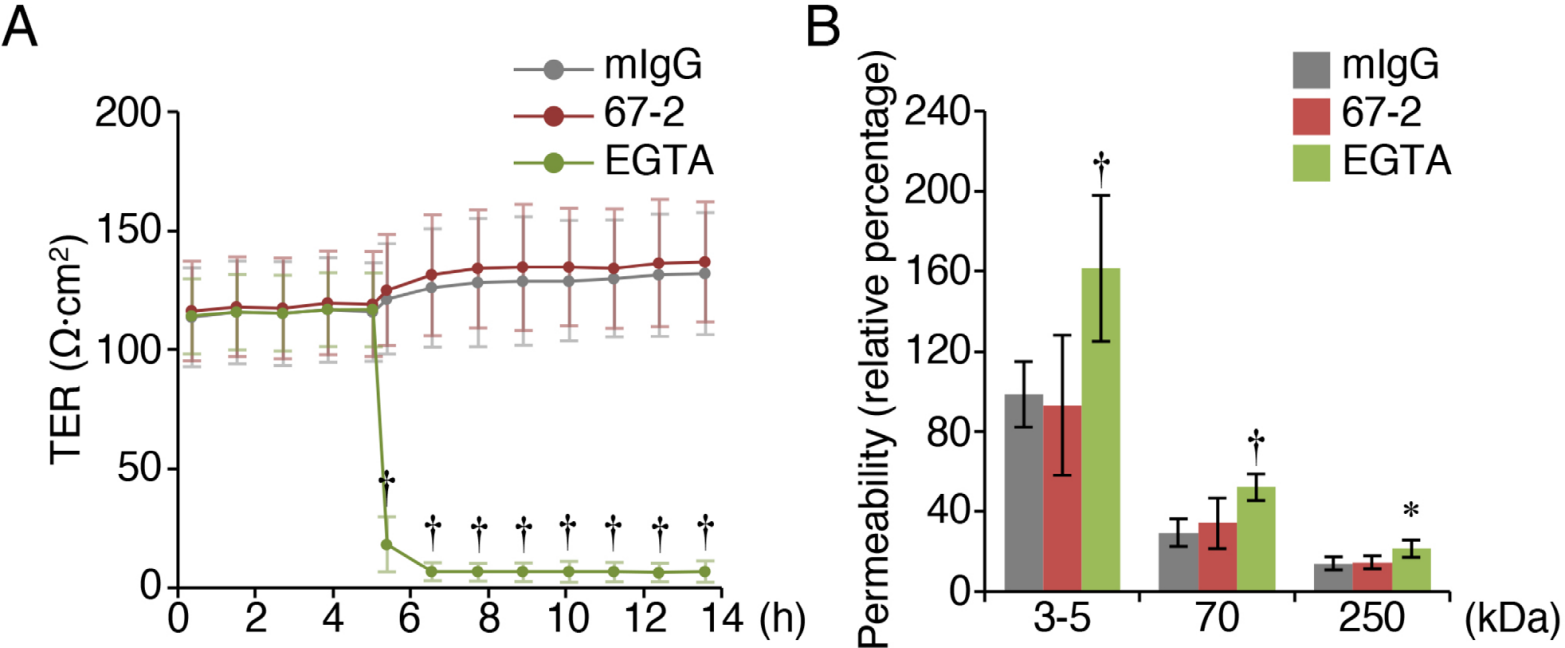
The mAb 67-2 does not alter the barrier function of tight junctions in Eph4 cells (**A, B**) Eph4 cells grown on the transwell inserts were treated with mIgG (50 μg), 67-2 (50 μg) or EGTA (2 μM), and transepithelial electrical resistance (TER) and permeability of tracers with indicated molecular weight were measured. All values represent the mean ± SD (error bars; A, *n* = 4; B, *n* = 8). Statistic values were determined compared with those in the mIgG-treated cells; ^*^P < 0.01, ^†^*P* < 0.001.

To evaluate whether the mAb 67-2 affects the barrier function of tight junctions, we measured transepithelial electrical resistance (TER) and permeability in mouse Eph4 cells, which are well-polarized and exhibit relatively high barrier function, grown on transwell inserts. The TER values were not altered in cells exposed to 67-2, but markedly declined in EGTA-treated cells (Figure 7A). Similarly, the permeabilities of 3–5, 70 and 250 kDa-tracers were significantly increased by EGTA treatment, but not by 67-2 exposure (Figure 7B).

## DISCUSSION

In the present study, we developed the mAb 67-2 against hOCLN-EC2, and demonstrated that basolaterally-applied mAb strikingly avoided HCV infection *in vitro*. This conclusion was drawn from three distinct culture systems of the HCV-permissive cell line Huh7.5.1. We showed that 67-2, upon basolateral addition to a double chamber-culture system, prevented HCVcc infection. We also indicated that the basolaterally-applied mAb 67-2 inhibited three different genotypes of HCVcc (genotype 2a), HCVpv (genotype 1a) and HCVpp (genotypes 1b and 2a) infection in a matrigel-embedded 3D culture system. Since matrigel-embedded Huh7 cells show hepatic polarity with bile canaliculi-structure [24], they seem to mimic HCV infection and prevention *in vivo*. By contrast, in Huh7.5.1 monolayers, the apically-applied mAb 67-2 barely blocked HCVcc and HCVpv infection, and prevented HCVpp infection less than anti-CD81 mAb. Taken together, our results indicate that the anti-hOCLN-EC2 mAb 67-2 is accessible to OCLN from the basolateral side of hepatocytes but not from the apical side. Effectiveness of 67-2 via the basolateral route suggests that it could be a promising anti-HCV agent.

**Figure 8:**
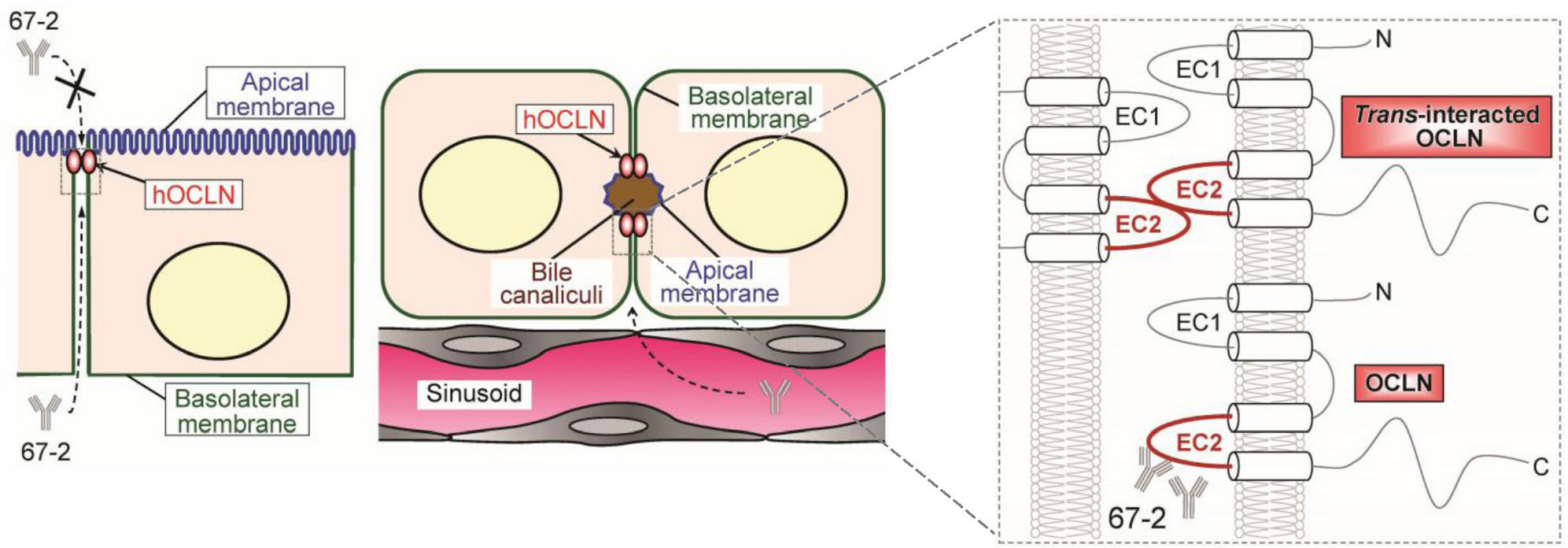
A schematic model for a proposed action mechanism of the mAb 67-2 The 67-2 is accessible to and eliminate unengaged basolateral OCLN through inhibiting oligomerization of OCLN irrespective of the presence or absence of HCV, thereby preventing the entry.

We designed the polypeptide antigen including four human-specific amino acids (A214, L215, A223 and A224) that are different from the mouse. However, the established 67-2 mAb reacted with not only human OCLN, but also mouse OCLN. Therefore, it is strongly suggested that 67-2 does not recognize these human-selective amino acids. In other words, the epitope of 67-2 is likely to be in either CNQFYTP (216-222) or TGLYVD (225-230). Interestingly, a single substitution of C216 with alanine in OCLN-EC2 hinders not only *cis*-interaction but also *trans*-interaction between plasma membranes of opposing cells, and reduces the membrane pool of OCLN [25]. In addition, an early study on OCLN strongly suggests that OCLN is first targeted to basolateral membranes from the cytoplasm and concentrated into tight junctions [26]. Taken collectively, we speculate that the mAb 67-2 is accessible to and eliminate unengaged basolateral OCLN through inhibiting the C216-based oligomerization of OCLN irrespective of the presence or absence of HCV, thereby preventing the entry (Figure 8).

The most established function of tight junction represents the barrier function to regulate the molecular penetration of ions, solutes and cells. The barrier function enables the epithelial layer to separate the body compartments from the external environment, and maintains homeostasis. Therefore, it is critical to determine whether antibodies or synthetic drugs targeting tight junction proteins exhibit adverse effects on the barrier function. Along this line, we showed, by measuring TER and permeability, that 67-2 did not alter the barrier function of tight junctions in the well-polarized epithelial cell line Eph4. This is reasonable because the barrier function of tight junctions is primarily determined by claudin family members but not by OCLN [27–31]. In fact, unlike claudins, OCLN is not capable of forming tight junction strands [30]. In addition, OCLN-null embryonic stem cells differentiate into polarized epithelial cells bearing well-developed tight junctions with no barrier dysfunction [32]. However, it should be mentioned that OCLN-deficient mice possess complex phenotype, suggesting an unexpected role of OCLN [33].

In summary, we have developed the anti-hOCLN-EC2 mAb 67-2 to prevent HCV infection *in vitro* with no adverse effects on cell viability or functions of tight junctions. Nevertheless, it should be determined whether 67-2 inhibits HCV infection without unfavorable effects *in vivo*. It should be also clarified how 67-2 inhibits HCV infection in future experiments. Since the mAb 67-2 reveals a unique property with accessibility to OCLN from the basolateral side but not from the apical side of cells, it would provide a powerful tool for elucidating the precise role of OCLN *in vitro* and *in vivo*.

## MATERIALS AND METHODS

### Generation of mouse anti-hOCLN-EC2 mAbs

To generate mouse mAbs against hOCLN-EC2, the peptide ALCNQFYTPAATGLYVD (aa 214-230), in which a cysteine residue was added at the N-terminal end, was used as an antigen. It was coupled via cysteine to keyhole limpet hemocyanin. The antigen was subcutaneously injected into mice four times every two weeks. B cells isolated from the spleen and mouse myeloma P6 cells were fused to make hybridomas. The culture supernatants of the hybridomas were subcloned by the limiting dilution technique, then screened by the ELISA-based binding assay to the antigenic peptide and by the inhibition of HCV infection. Finally, mAbs from the culture supernatant of the subclones were purified using Ab-Rapid PuRe affinity gel (Protenova, Kagawa, Japan) and gentle Ag/Ab binding and elution buffer (Thermo Fisher Scientific, Waltham, MA).

### Cell culture

The HCV-permissive human cell line Huh7.5.1 was obtained from the American Type Culture Collection (Rockville, MD). The well-polarized mouse mammary epithelial cell line Eph4 and the African green monkey kidney-derived cell line COS-7 cells were kindly provided by Prof. Mikio Furuse (National Institute for Physiological Sciences, Okazaki, Japan) and Prof. Pierre Chambon (Institut de Génétique et de Biologie Moléculaire et Cellulaire, Illkirch-Graffenstanden, France), respectively. The Huh7.5.1 and COS-7 cells were cultured in D-MEM high glucose (Sigma, St. Louis, MO) with 10% FBS (Thermo Fisher Scientific) at 37°C in 5% CO_2_. The Eph4 cells were grown in D-MEM high glucose (Sigma) with 10% FBS and 1% penicillin/streptomycin (Thermo Fisher Scientific) at 37°C in 5% CO_2_. The hybridoma cells were cultured in RPMI (Wako, Osaka, Japan) with 20% FBS and 1% penicillin/streptomycin at 37°C in 5% CO_2_.

### Evaluation of inhibition of HCVcc infection

To prepare the Huh7.5.1 monolayer, 1 × 10^5^ cells were plated on a 24-well flat-bottom plate, and incubated for 48 h. After exchange of media, they were treated with either of the following: medium alone (mock group); 20 μg of normal mouse IgG (Jackson ImmunoResearch, West Grove, PA); 2.5 μg of anti-CD81 antibody (JS-81 clone, 555675, BD Biosciences Pharmingen, Mountain View, CA); or 100 μl of culture medium from each antibody-producing clone. For the Huh7.5.1-double chamber culture system, 1 × 10^4^ cells were seeded on cell culture insert (cellulose mixed ester, 24-well, pore size 0.4 μm, translucent, BD Falcon, Flanklin Lakes, NU), and incubated for 48 h. After changing the media, they were exposed to either of the following: medium alone (mock); 50 μg of normal mouse IgG; 2.5 μg of anti-CD81 antibody; or 50 μg of anti-hOCLN-EC2 mAbs in the external chamber. For the Matrigel-embedded 3D culture system of Huh7.5.1, cells were prepared using the method described by Molina-Jimenez *et al*. [24], with slight modifications. Fifty μl of Matrigel (Corning, Tewksbury, MA) was added to a 48-well flat-bottom plate, and 5 × 10^3^ cells were plated and grown for six days. They were then incubated with either of the following: medium alone (mock); 50 μg of normal mouse IgG; 2.5 μg of anti-CD81 antibody; or 50 μg of the anti-hOCLN-EC2 mAb 67-2.

One hour after treatment, HCVcc derived from a JFH-1 strain (cell cultured HCV; genotype 2a) [34] was apically (monolayer and double chamber culture system) or basolaterally (3D culture system) inoculated to the cells, and incubated for 2 h. They were subsequently washed twice in the medium and cultured for 24 h, followed by RNA extraction and the detection of HCV RNA by real-time PCR

### Evaluation of inhibition of HCVpv and HCVpp infection

Pseudotype HCV virus (HCVpv), a vesicular stomatitis virus (VSV) covered with HCV envelope proteins, con1 (genotype 1a), and a negative control VSV integrated GFP gene without HCV envelope proteins, were used [34]. HCV pseudo-particles (HCVpp), generated by assembling unmodified E1 and E2 glycoproteins derived from JFH1 (genotype 2a) and TH (genotype 1b) onto retroviral core proteins derived from MLV, were also used [34]. In addition, VSV pseudo-particles (VSVpp) containing VSV G glycoprotein were produced as negative controls [34]. The Huh7.5.1 cells were grown either in a 48-well flat-bottom plate for one day, or in matrigel-coated 48-well flat-bottom plate (3D culture system) for six days. They were incubated for 1 h with either of the following: medium alone (mock); 50 μg of normal mouse IgG; 2.5 μg of anti-CD81 antibody; or 50 μg of the anti-hOCLN-EC2 mAb 67-2. Following incubation, the cells were treated for 48 h with HCVpv, VSVpv, HCVpp and VSVpp, and the luciferase activity was measured by using the Luciferase Assay System (Promega, Madison, WI). In brief, after removing the medium, 100 μl of lysate was added and left for 15 min. Twenty μl of lysates were collected, and mixed with 50 μl of luciferase substrate before measurement using a luminometer (Promega) after vortex stirring. A decrease of ≥90% compared to the mock group was defined as an inhibitory effect on infection.

### Real-time PCR

Reverse transcription and polymerase chain reaction (PCR) were performed on a Step One Real-Time PCR system (Applied Biosystems, Foster City, CA) using a TaqMan^®^ One-Step RT-PCR Master Mix Reagents Kit (Toyobo, Tokyo, Japan). Reverse transcription was performed at 90°C for 30 s, at 61°C for 20 min, and at 95°C for 1 min, and PCR was performed for 45 cycles of 15 s at 95°C and 1 min at 60°C. The PCR primer and probe sequences were as follows: NS5A: forward 5′-AGT ACC ACA AGG CCT TTC G-3′, reverse 5′-CGG GAG AGC CAT AGT GG-3′; Taqman probe: 5′-(FAM)-CTG CGG AAC CGG TGA GTA CAC-(TAMRA)-3′.

### Immunofluorescence staining

Matrigel (50 μl/well) was added to an 8-well glass chamber plate, and 5 × 10^3^ Huh7.5.1 cells were plated with 200 μl of culture medium, followed by six days of culture. They were exposed for 1 h to either medium alone (mock) or the mAb 67-2 (50 μg/well), and grown for an additional 1 h in the presence or absence of HCVcc (5 μl/well). The cells were then washed with PBS, and fixed for 10 min in −20°C-cold methanol before blocking with 2% BSA/PBS at room temperature for 1 h. The cells were incubated for 2 h at room temperature with rabbit anti-NS5A antibody [35] that was diluted 200-fold in Signal Booster (Beacle, Kyoto, Japan). They were subsequently reacted with donkey anti-rabbit IgG-Alexa Fluor 488 (Molecular Probes) and DAPI (Dojindo, Kumamoto, Japan), immunofluorescence images were observed using confocal laser scanning microscopy (FV-1000, Olympus, Tokyo, Japan).

### Evaluation of cytotoxicity

A XTT cell proliferation assay kit II (Roche Diagnostics, Mannheim, Germany) was used to evaluate the cytotoxicity of the anti-hOCLN-EC2 mAb 67-2 in 3D culture of the Huh7.5.1 cells. Matrigel (25 μl/well) was added to a 96-well flat-bottom plate, and 5 × 10^3^ cells were seeded with 100 μl of culture medium, followed by six days of culture. They were treated for 1 h with either of the following: medium alone (mock); normal mouse IgG; or the mAb 67-2. To evaluate the effects of HCV infection, HCVcc (10 μl/well) was added 1 h after the addition of medium alone (mock), 50 μg/well of normal mouse IgG or the mAb 67-2, and cells were grown for 24 h.

### Immunoblot analysis

Mouse liver, kidney and cerebral cortex tissues as well as Huh7.5.1 cells were lysed with RIPA buffer (50 mM Tris-HCl [pH 7.4], 150 mM NaCl, 1% NP40, 0.1% SDS, 0.5% DOC, 1 mM PMSF, 5 mM EGTA) containing a protease inhibitor cocktail (Roche Diagnostics). Total cell lysates were resolved by SDS-polyacrylamide gel, and electroblotted onto an Immobilon-P PVDF membrane (Millipore, Belford, MA, USA). The membrane was saturated with TBS containing 5% nonfat dry milk and 0.1% Tween 20, then incubated overnight at 4°C with 67-2 in TBS containing 1% skimmed milk. After rinsing in TBS containing 0.1% Tween 20, the membrane was incubated for 1 h at room temperature with horseradish peroxidase-conjugated anti-mouse IgG (GE healthcare, Buckinghamshire, UK) in TBS containing 1% nonfat dry milk. It was then rinsed again, and finally reacted using ECL prime western blotting detection reagent (GE Healthcare). The blots were stripped and immunoprobed with a mouse mAb against the C-terminal domain of hOCLN (33-1500, Invitrogen, Carlsbad, CA, USA) and a mouse mAb against β-actin (A5441; Sigma). The signals were visualized with an ImageQuant LAS4000 system (GE Healthcare). The animal experiment was approved by the Experimental Animal Center of Fukushima Medical University and carried out in accordance with regulations on animal experiments.

### Isotype analysis of the antibody

IsoStrip Mouse Monoclonal Antibody Isotyping Kit (Roche Diagnostics) was used for the analysis. Isotype strips were immersed after instilling 1 mg/ml of the mAb 67-2 into a tube and stirring. After standing for 5 min, the isotype was determined based on the blue band on the strip.

### Measurement of barrier function

1 × 10^5^ Eph4 cells were seeded onto the transwell inserts (cellulose mixed ester, 24-well, pore size 0.4 μm, translucent, BD Falcon), and cultured for four days. After TER values had reached a plateau, media were changed from D-MEM high glucose to Opti-MEM (Thermo Fisher Scientific), and 50 μg of mouse IgG or the mAb 67-2 were added to the lower chamber. 2 mM EGTA (Sigma) was applied to the upper chamber. TER value was measured with cellZscope instrument (CellSeed, Tokyo, Japan).

For the paracellular tracer flux assay, 10 min after applying mouse IgG, 67-2 or EGTA, 1 mg/ml fluorescein isothiocyanate (FITC)-labeled dextran with the molecular mass of 3-4 kDa, 70 kDa and 250 kDa (Sigma) was added to the upper chamber. After incubation for 2 h, the concentration of each tracer in the lower chamber was determined by measuring fluorescence with microplate reader (Varioskan, Thermo Fisher Scientific).

### Statistical analysis

Statistical significance of differences was evaluated by the Student's *t*-test and the Mann–Whitney *U* test for evaluation of HCVcc infection inhibition, with *p* < 0.05 considered to indicate statistical significance.
